# Oral-predominant microbial and functional dysbiosis in craniofacial microsomia: species-level profiling with compartment-matched statistical testing

**DOI:** 10.1080/20002297.2026.2754580

**Published:** 2026-11-03

**Authors:** Zhifeng Li, Tianying Zang, Zhiyong Zhang, Wei Liu, Lin Yin, Shanbaga Zhao, Bingyang Liu, Lunkun Ma, Xiaojun Tang

**Affiliations:** a Center for Plastic & Reconstructive Surgery, Department of Plastic & Reconstructive Surgery, Zhejiang Provincial People's Hospital (Affiliated People's Hospital), Hangzhou Medical College, Hangzhou, Zhejiang, China; b Department of Aesthetic Plastic Surgery and Laser Medicine, Beijing Anzhen Hospital Affiliated to Capital Medical University, Beijing, China; c Department of Comprehensive Surgery, Plastic Surgery Hospital, Chinese Academy of Medical Sciences, Peking Union Medical College, Beijing, China; d Department of Maxillofacial Surgery, Plastic Surgery Hospital, Chinese Academy of Medical Sciences, Peking Union Medical College, Beijing, China

**Keywords:** Craniofacial microsomia, oral microbiome, 2bRAD-M, alveolar bone loss, species-level profiling, compartment-matched analysis

## Abstract

**Background:**

Craniofacial microsomia (CFM) is the second most common congenital craniofacial anomaly. Oral dysbiosis has been described at genus level, but whether it extends beyond the mouth, or relates to the defining alveolar bone deficiency, is unknown.

**Patients/materials and methods:**

Oral and gut microbiomes of 45 children with CFM and 18 matched controls were profiled at species level by 2bRAD-M, alongside shotgun metagenomes and blood, faecal and oral metabolomes. Alveolar bone loss was quantified on computed tomography against each patient's contralateral side. Centred-log-ratio methods with Benjamini-Hochberg false-discovery-rate (FDR) control were used throughout. Compartment specificity was tested formally by a group-by-compartment interaction model in the 58 participants with paired oral and gut profiles, by sample-size-matched subsampling, and by minimum-detectable-effect calculation.

**Results:**

Oral alpha diversity was higher in CFM than in controls (Shannon *P* = 0.014; all three alpha-diversity indices *P* < 0.05). Four species-level taxa were depleted after false-discovery-rate (FDR) correction (q < 0.05), and 21 interpretable metagenomic pathways differed between groups among 38 FDR-significant pathways. These annotations were enriched for carbohydrate transport, glycan processing and cell-surface or biofilm-related functions, but they are not specific markers of a cariogenic or pathogenic community. No FDR-robust gut or systemic signature was detected. At the species level, the oral and gut analyses were power-matched (minimum detectable |r| = 0.74 at the FDR-equivalent threshold), and the group-by-compartment interaction for Shannon diversity was significant (*P* = 0.026). This comparison supports an oral-predominant taxonomic pattern but does not establish biological absence of a gut effect. The functional comparison was not power-matched. In the patient-only post hoc analyses, the oral functional pattern was not associated with tooth-brushing frequency, sugary-drink intake or caries burden. Affected-side alveolar bone density was lower than contralateral density (paired *P* < 0.001) and was not associated with anatomical severity grade.

**Conclusions:**

CFM was associated with an oral-predominant taxonomic and functional microbiome pattern. The matched-power comparison is informative for taxonomic analyses but does not exclude a biologically meaningful gut effect, and the functional comparison remains underpowered. These observations motivate prospective investigation of local, biofilm-directed interventions and of quantitative alveolar bone assessment as a follow-up endpoint.

## Key messages

- In craniofacial microsomia, species-level profiling identifies oral taxonomic and functional differences. At the species level, oral and gut analyses were power-matched, and a group-by-compartment interaction supports an oral-predominant pattern; this statistical comparison does not establish biological absence of a gut effect.
- Quantitative alveolar bone loss is lower on the affected side and is decoupled from the anatomical severity classifications assessed in this cohort.
- The cross-sectional findings motivate, but do not support, prospective testing of local, biofilm-directed interventions; they also identify quantitative alveolar bone assessment as a measurable follow-up endpoint in craniofacial microsomia.

## Introduction

Craniofacial microsomia (CFM) is the second most common congenital craniofacial anomaly after orofacial clefting, arising from disrupted development of the first and second pharyngeal arches and presenting with variable hypoplasia of the mandible, ear, facial nerve and associated soft tissues [1,2]. Deficiency of the alveolar bone is a defining feature that complicates dental development, orthodontics and orthognathic reconstruction, yet it is seldom quantified: clinical assessment has traditionally relied on anatomical, largely static staging systems such as the Pruzansky-Kaban classification and the OMENS/OMENS-Plus scheme [3,4]. These schemes capture skeletal and soft-tissue morphology but not the microbial and metabolic dimensions of the disorder, and the biological factors that accompany alveolar bone deficiency in CFM remain poorly understood.

The human microbiome is increasingly recognised as a regulator of host skeletal, immune and metabolic biology. In the oral cavity, resident communities modulate alveolar bone homeostasis through osteoimmune signalling, and germ-free animals exhibit markedly altered alveolar bone remodelling [5,6]. Children with craniofacial anomalies are predisposed to oral ecological variation through structural, feeding and hygiene-related factors [7], and a previous saliva-based survey reported altered diversity and selected taxonomic differences in CFM [8]. That work, however, was confined to the oral cavity and to 16S profiling with a small metagenomic subset. Whether the observed oral pattern extends to the gut microbiome or to circulating and faecal metabolomes, as has been proposed for the oral-gut-systemic axis in periodontitis [9], remains unknown in CFM.

Addressing these questions requires simultaneous, compartment-resolved profiling rather than isolated single-omic comparisons, together with a statistically disciplined analytic strategy: multi-omics data are high-dimensional, compositional and prone to inflated associations, so compositional transformation, false-discovery-rate control, and a clear separation of confirmatory from exploratory findings are essential [10,11]. A conservative, association-level framing is particularly important for a cross-sectional design, in which directionality and mechanism cannot be established.

Here we assembled a multi-omics cohort of 45 patients with CFM and 18 matched controls, integrating species-level oral and gut microbiomes (2bRAD-M) [12], oral shotgun metagenomes, and blood and faecal metabolomes, with intra-individual computed-tomography quantification of alveolar bone loss. We aimed to (i) characterise oral taxonomic and functional differences in CFM; (ii) test whether the observed oral pattern is also detectable in the gut microbiome and circulating or faecal metabolomes; and (iii) quantify alveolar bone loss against the contralateral side and determine its relation to anatomical staging. We use compositional transformation and false-discovery-rate control, report the tested feature universe, and distinguish FDR-controlled findings from analyses that are underpowered for a cross-compartment comparison. Relative to the earlier genus-level survey, this study provides species-level taxonomic resolution, a larger cohort, simultaneous profiling across oral, gut and metabolomic compartments, and a quantitative, intra-individually controlled imaging measure of alveolar bone loss.

## Patients, materials and methods

### Study design and participants

This observational, cross-sectional study enrolled 63 participants: 45 patients with craniofacial microsomia (CFM) and 18 age- and sex-matched controls without craniofacial anomaly or systemic disease, drawn from patients undergoing minor elective scar-revision surgery, recruited between October 2022 and April 2023 at the Plastic Surgery Hospital, Chinese Academy of Medical Sciences, Peking Union Medical College. Age and sex did not differ significantly between groups (Table 1). Patients were classified into clinical subtypes (IIa, IIb, III) by the Pruzansky-Kaban system based on mandibular and temporomandibular-joint morphology [3], and overall severity was graded with the OMENS-Plus scheme across the orbital, mandibular, ear, facial-nerve and soft-tissue domains [4]. Inclusion criteria were: (1) a definitive diagnosis of CFM (cases) or absence of systemic disease (controls); (2) age 6–12 years (mixed-dentition stage); and (3) written informed consent from a parent or guardian, with age-appropriate assent. To limit microecological confounding, participants were excluded for other systemic diseases or congenital syndromes; probiotics, topical fluoride treatment or antibiotics within 3 months; periodontal or obstructive-sleep-apnoea treatment within 1 month; specific dietary preferences (for example vegetarianism); pet ownership; or acute discomfort on the sampling day. Because collection and platform-specific quality control differed by data layer, analytic sample sizes varied: oral and faecal microbiomes were profiled by 2bRAD-M (oral *n* = 63; faecal *n* = 58); oral and faecal shotgun metagenomes were obtained for subsets (oral *n* = 48 [34 CFM, 14 controls]; faecal *n* = 22 [13 CFM, 9 controls]); and blood and faecal metabolomes were acquired (blood *n* = 43 [31 CFM, 12 controls]; faecal *n* = 22). The oral metabolome was not used for case-control or cross-compartment inference because of exogenous dexmedetomidine contamination. The aggregate participant and analytic flow is shown in Figure 1A; per-participant data availability is shown in Supplementary Figure S5. The protocol was approved by the Institutional Review Board of the Plastic Surgery Hospital, Chinese Academy of Medical Sciences, Peking Union Medical College (approval no. 2021–199), and conducted in accordance with the Declaration of Helsinki. Review of the clinical records confirmed that no patient had undergone mandibular distraction osteogenesis, alveolar bone grafting or another procedure involving the oral skeleton before computed tomography, so the bone-density measurements reflect the untreated state.

**Table 1.** Demographic and clinical characteristics of the study cohort.

Characteristic	Total (*N* = 63)	CFM (*N* = 45)	Control (*N* = 18)	P
Age, years [*n* = 63]	8.29 ± 1.65	8.44 ± 1.66	7.89 ± 1.60	0.236
Sex, *n* (%)				0.783
Male	34 (54.0%)	25 (55.6%)	9 (50.0%)	
Female	29 (46.0%)	20 (44.4%)	9 (50.0%)	
C-reactive protein, mg/L [*n* = 35]	—	1.13 ± 1.04	—	—
Interleukin-6, pg/mL [*n* = 35]	—	3.22 ± 2.06	—	—
White blood cells, 10^9/L [*n* = 35]	—	6.62 ± 1.90	—	—
Alveolar bone loss—inner plate, HU [*n* = 35]	—	203.11 ± 201.52	—	—
Alveolar bone loss—outer plate, HU [*n* = 35]	—	162.23 ± 257.28	—	—
Total alveolar bone loss, HU [*n* = 35]	—	365.34 ± 380.20	—	—
OMENS-Plus total score, points [*n* = 45]	—	6.97 ± 1.80	—	—
Pruzansky-Kaban type, *n* (%) [*n* = 45]	—		—	—
I	—	0 (0.0%)	—	
IIa	—	22 (48.9%)	—	
IIb	—	21 (46.7%)	—	
III	—	2 (4.4%)	—	
OMENS-M grade, *n* (%) [*n* = 45]	—		—	—
1	—	1 (2.2%)	—	
2A	—	11 (24.4%)	—	
2B	—	31 (68.9%)	—	
3	—	2 (4.4%)	—	
OMENS—Orbital (O), *n* (%) [*n* = 45]	—		—	—
0	—	32 (71.1%)	—	
1	—	6 (13.3%)	—	
2	—	6 (13.3%)	—	
3	—	1 (2.2%)	—	
OMENS—Ear (E), *n* (%) [*n* = 45]	—		—	—
0	—	1 (2.2%)	—	
1	—	1 (2.2%)	—	
2	—	37 (82.2%)	—	
3	—	6 (13.3%)	—	
OMENS—Facial nerve (*N*), *n* (%) [*n* = 45]	—		—	—
N0	—	25 (55.6%)	—	
N1	—	9 (20.0%)	—	
N2	—	4 (8.9%)	—	
N3	—	7 (15.6%)	—	
OMENS—Soft tissue (S), *n* (%) [*n* = 45]	—		—	—
0	—	7 (15.6%)	—	
1	—	22 (48.9%)	—	
2	—	15 (33.3%)	—	
3	—	1 (2.2%)	—	
Trigeminal (V) involvement, *n* (%) [*n* = 45]	—		—	—
Yes	—	2 (4.4%)	—	
No	—	43 (95.6%)	—	
Hypoglossal (XII) involvement, *n* (%) [*n* = 45]	—		—	—
Yes	—	12 (26.7%)	—	
No	—	33 (73.3%)	—	
Extracranial anomalies (OMENS-Plus), *n* (%) [*n* = 45]	—		—	—
Present	—	4 (8.9%)	—	
Absent	—	41 (91.1%)	—	

Data are mean ± SD or n (%). Age compared by Wilcoxon rank-sum test; sex by Fisher's exact test.

Variables measured in patients only are shown as ‘—’ in the Total and Control columns, and their percentages use the number of patients in whom the variable is defined as the denominator.

The number of participants contributing to each variable is given in square brackets; note that CRP, IL-6, white-cell count and all alveolar bone-loss measures were available in 35 of the 45 patients.

Pruzansky-Kaban and OMENS-M are independent mandibular classification systems and are reported separately.

HU, Hounsfield units.

**Figure 1.**
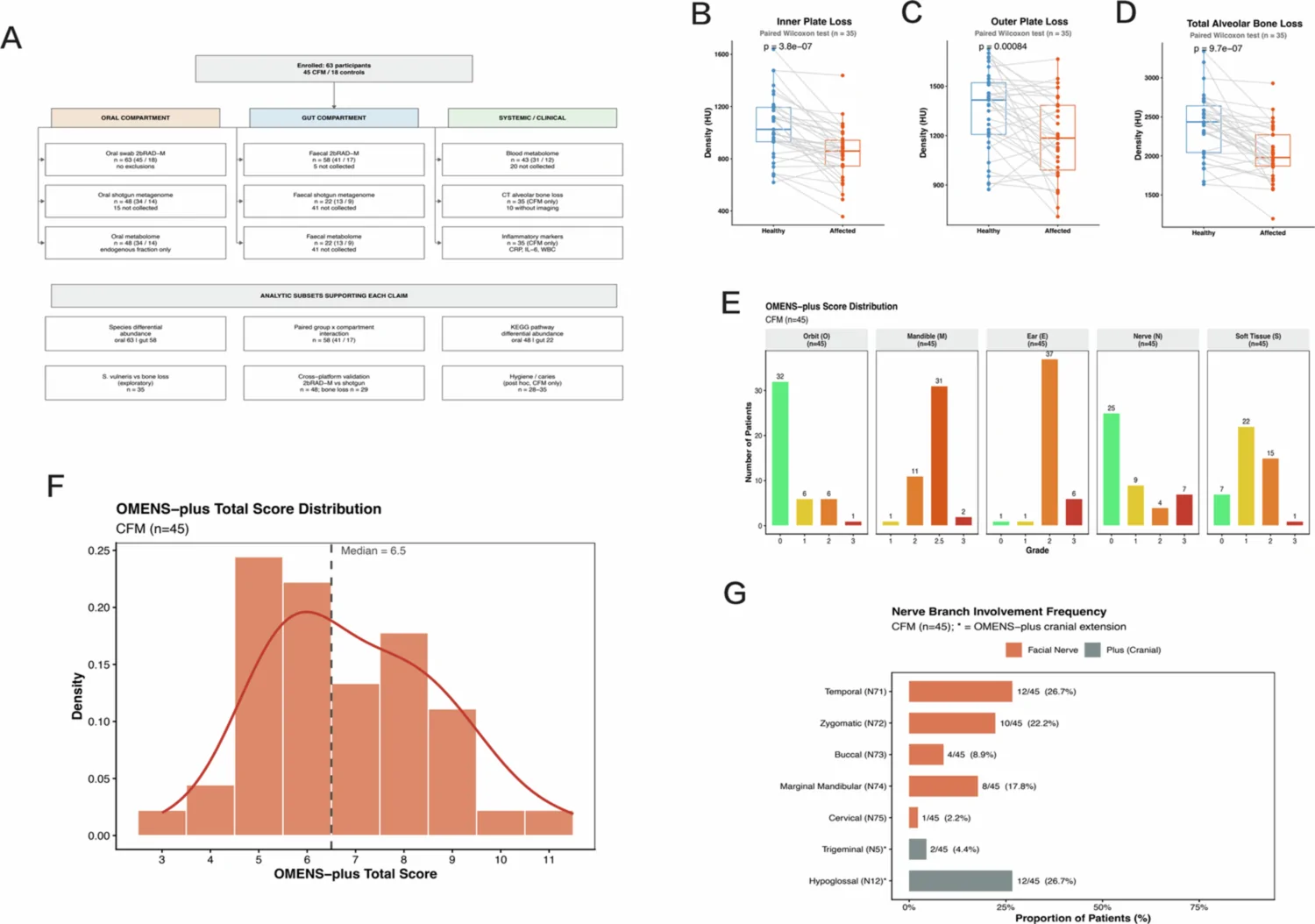
Cohort composition, multi-omics data availability and quantitative alveolar bone loss in craniofacial microsomia (CFM). (A) Participant and analytic-sample flow from 63 enrolled participants (45 CFM, 18 controls) through sample collection and platform-specific quality control. (B–D) Paired comparison of alveolar bone density (Hounsfield units, HU) between affected and contralateral sides in CFM patients with complete imaging (*n* = 35), shown for the inner cortical plate (B), outer cortical plate (C) and total alveolar bone (D). Grey lines connect measurements from the same individual; *P* values are from two-sided paired Wilcoxon signed-rank tests. (E) OMENS-Plus grades across the orbit, mandible, ear, facial-nerve and soft-tissue domains in CFM patients (*n* = 45). (F) Distribution of the OMENS-Plus total score. (G) Frequency of facial-nerve branch involvement and OMENS-Plus cranial-nerve extension (*n* = 45). Full imaging statistics are in Table S1.

### Clinical phenotyping and imaging-based quantification of alveolar bone loss

Alveolar bone density was quantified from 128-slice multidetector (spiral) computed tomography (Siemens Healthineers, Germany) in a subset of CFM patients (*n* = 35). Regions of interest were delineated on the affected and contralateral alveolar plates and mean density (Hounsfield units, HU) computed in Mimics (Materialise, Leuven, Belgium). For each patient an intra-individual paired comparison used the contralateral side as an internal control; absolute bone loss of the inner and outer cortical plates was defined as the HU deficit on the affected side. A composite Total Alveolar Bone Loss was defined as (healthy inner HU + healthy outer HU) - (affected inner HU + affected outer HU). Investigators delineating regions of interest and computing HU were blinded to all multi-omics data. To test whether bone loss tracked categorical anatomical severity, bone-loss measures were compared across OMENS-M grades and Pruzansky-Kaban types by Kruskal-Wallis test and correlated with the OMENS-Plus total score (Spearman); non-parametric tests were used because bone-loss values deviated from normality (Supplementary Figure S1; Table S1).

#### Oral-hygiene, dietary and dental variables (post hoc)

Oral-hygiene behaviour, dietary and dental items were recorded on the enrolment case report form but were not part of the pre-specified analysis plan; they were therefore analysed post hoc. These items were recorded for patients only, so no case-control comparison was possible and all analyses are within-patient. The instrument was not a validated questionnaire. Tooth-brushing was recorded as free text (for example '2 times x 20 s') and coded into frequency (times per day) and duration (seconds per episode). Frequency dichotomised at > = 2 per day was pre-specified as the primary exposure, because the frequency of mechanical disruption rather than cumulative brushing time governs dental-biofilm maturation, and because frequency and duration were inversely related in this cohort (Spearman rho = −0.23), so that their product would have obscured rather than summarised the contrast; duration was treated as a secondary exposure and the daily product as a sensitivity analysis. Additional items were post-meal rinsing (episodes per day), sugary or carbonated drink intake (0 = almost never to 3 = >= 5 times per week), caries count, and reported gingival bleeding or recurrent oral ulceration. Items with no usable variance (separate toothbrush storage, all participants positive; dental floss or toothpick use, 32 of 35 negative; vegetable and fruit intake, *n* = 11 and uniform) were not analysed. Availability ranged from *n* = 28 to *n* = 35; all variables, their distributions and the reasons for exclusion are listed in Table S6.

### Biological sample collection

Multi-compartment samples were collected to capture local, distal and systemic layers. Oral swabs: participants refrained from tooth-brushing for 24 h and from food or drink for 1 h, rinsed with 20 mL physiological saline for 1 min, and sterile cotton swabs were then held in the mouth for 2 min. Faecal samples: fresh morning stool was collected on the ward, kept at 4 C during transport and snap-frozen in liquid nitrogen within 2 h to prevent ex vivo compositional drift. Peripheral blood: fasting venous blood was drawn into EDTA tubes, gently inverted, and plasma separated by centrifugation (3000 rpm, 10–15 min, 4 C) within 30 min. All samples were stored at −80 C until extraction.

### Microbiome profiling: 2bRAD-M and shotgun metagenomics

Microbial genomic DNA was extracted from oral swabs and faeces with the MagPure Soil DNA KF Kit; concentration and integrity were assessed on a NanoDrop 2000 (Thermo Fisher Scientific). Sequencing and primary processing were performed by OE Biotech (Qingdao/Shanghai, China). To avoid confounding between technical batch and case-control status, each compartment-platform combination was processed as a single extraction, library-preparation and sequencing batch, with patients and controls interleaved within the batch rather than processed in group-wise blocks (2bRAD-M: oral batch *n* = 63, faecal batch *n* = 58; shotgun metagenomics: oral batch *n* = 48, faecal batch *n* = 22). No library preparation failed. Batch was therefore not testable as a covariate but is by construction orthogonal to group assignment within each compartment; because each compartment was processed as a single batch, no cross-batch correction (for example ComBat) was applicable. No conclusion depends on cross-batch normalisation, because every comparison is of case-control effect sizes computed within a compartment rather than of raw abundances across compartments; a batch effect, if present, would act equally on patients and controls processed in the same batch. Sequencing depth did not differ between groups in either compartment (oral *P* = 0.22, faecal *P* = 0.60), and in a multivariable PERMANOVA the group effect on oral community structure persisted after adjustment for sequencing depth, age and sex (R2 = 0.022, *P* = 0.041). Direct cross-compartment contrasts of absolute abundance remain descriptive; where a difference between compartments is claimed, it is tested formally as a group-by-compartment interaction (Section 2.6.6).

#### 2bRAD-M species-resolved profiling

2bRAD-M libraries were prepared following Wang et al. with minor modifications [12,13]. DNA was digested with BcgI (NEB; 4 U, 37 C, 3 h), adaptors were ligated (T4 DNA ligase, 4 C, 12 h), products were amplified and size-selected (~100 bp) on 8% polyacrylamide gel, and sample-specific barcodes were introduced by PCR before sequencing on the Illumina NovaSeq (PE150) platform. For taxonomic assignment, species-specific 2bRAD tags were derived in silico from 404,199 microbial genomes (GTDB and Ensembl) digested with Type IIB enzymes to build a species-level marker database (86,022 species). Quality-controlled reads were mapped to this database, and false-positive species calls were controlled using the G-score (a harmonic mean of marker coverage and marker number) with a threshold of 5; species relative abundance was then computed from per-marker read coverage over a sample-specific candidate database [12]. Species nomenclature throughout this study therefore follows GTDB rather than NCBI taxonomy, including the post-R214 phylum names (for example Bacillota) and the GTDB generic placements (for example Phocaeicola, split from Bacteroides). Alphabetic suffixes on species names (for example Streptococcus mitis_I, Granulicatella elegans_A) denote distinct GTDB species clusters that subdivide the corresponding NCBI species; they are not synonyms of that species and should not be equated with it. Full seven-rank GTDB lineage strings for every taxon named in this article are given in Table S2 to allow unambiguous identification.

#### Shotgun metagenomics and functional annotation

Shotgun metagenomic sequencing (Illumina NovaSeq, PE150) was performed on oral (*n* = 48; 34 CFM, 14 controls) and faecal (*n* = 22; 13 CFM, 9 controls) samples. Reads were trimmed with Fastp v0.20.1 [14]; host reads were removed by alignment to the human genome with Bowtie2 v2.2.9 [15]; contigs were assembled with MEGAHIT v1.1.2 [16]; open reading frames were predicted with Prodigal v2.6.3 [17] and clustered into a non-redundant gene catalogue with CD-HIT v4.5.7 [18]. Taxonomy was assigned with DIAMOND v0.9.7 against the NR database, and functions were annotated against KEGG [19,20]. KEGG level-3 pathway abundances were summed from constituent KEGG Orthologs. Of 429 annotated pathways, 209 assigned to metabolism, selected transport and catabolism categories, and genetic-information processing were carried forward for testing; human-disease and organismal-system categories were excluded a priori as uninterpretable for a bacterial community. Pathways belonging to four KEGG level-2 categories that cannot be interpreted for an oral bacterial community (xenobiotics biodegradation and metabolism, biosynthesis of other secondary metabolites, metabolism of terpenoids and polyketides, and viral pathways) were flagged before interpretation as probable homology-driven annotation artefacts. This rule was applied to the complete tested pathway list in Table S3; flagged pathways are reported but not interpreted or displayed in Figure 2E.

**Figure 2.**
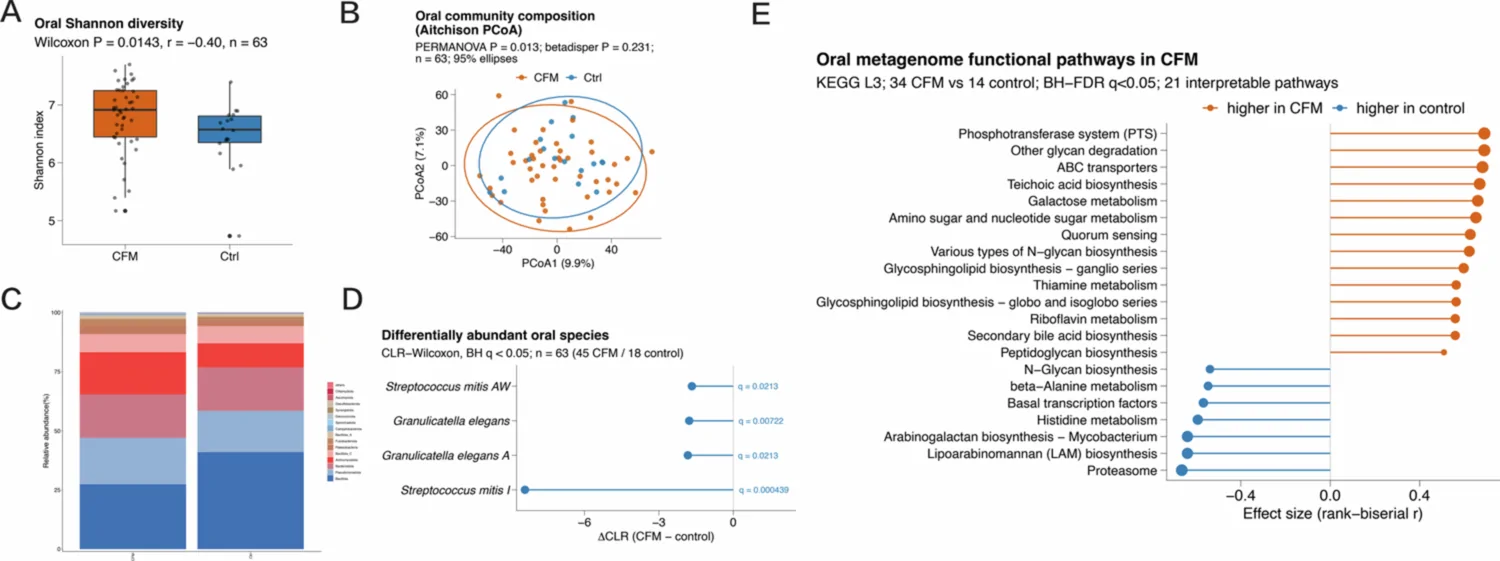
Oral microbiome differences in CFM. (A) Oral Shannon diversity in CFM versus controls; *P* is from a two-sided Wilcoxon rank-sum test and r is the rank-biserial correlation (*n* = 63; 45 CFM, 18 controls). (B) Principal coordinates analysis of Aitchison distance (Euclidean distance on centred-log-ratio-transformed abundance); ellipses are 95% confidence regions. *P* values are from PERMANOVA (999 permutations) and betadisper. (C) Descriptive phylum-level relative abundance in each group. (D) Differentially abundant oral species (CLR-Wilcoxon, BH-FDR q < 0.05); the x-axis is the difference in CLR-transformed abundance (CFM minus control), so negative values indicate lower abundance in CFM. (E) Twenty-one interpretable oral KEGG level-3 pathways that met BH-FDR q < 0.05 (34 CFM, 14 controls). Effect size is the rank-biserial correlation; orange denotes higher inferred pathway abundance in CFM and blue higher abundance in controls. Seventeen additional FDR-significant pathways in prespecified non-interpretable KEGG categories are reported in Table S3 but are not shown or biologically interpreted.

#### Sequencing yield, host content and negative controls

For 2bRAD-M, libraries were sequenced to a median of 7.5 × 10^6 raw reads per sample (oral, range 5.8–9.2 × 10^6; faecal, range 5.4–10.7 × 10^6), of which a median of 86.0% (oral) and 86.8% (faecal) were retained after enzyme-site recognition and quality filtering; 3,013 and 3,463 species were detected in total in the oral and faecal compartments, respectively. For shotgun metagenomics, sequencing yielded a median of 78.0 × 10^6 raw read pairs per oral sample (range 67.9–83.2 × 10^6) and 79.7 × 10^6 per faecal sample (range 73.4–83.3 × 10^6); quality trimming retained 99.7% and 99.9% of reads, respectively. Because oral swabs are dominated by host DNA, only a median of 9.8% of quality-filtered oral reads survived host removal, compared with 99.97% for faecal samples; the lower effective microbial depth of oral shotgun libraries is a limitation of swab-based metagenomics. Per-sample read counts, quality metrics and host-removal fractions are provided in Table S1. Extraction and library-preparation blanks were processed and sequenced with each 2bRAD-M run. Both yielded less than 0.05% of study-sample read depth. Recognised reagent-associated genera were undetectable in the study samples, whereas taxa abundant at the matching body site were consistent with trace well-to-well carryover rather than a reagent source. No mock-community positive control was included and no in-silico decontamination was applied; blank results were therefore used qualitatively rather than for statistical decontamination. No negative controls were included in the shotgun metagenomic runs.

### Untargeted dual-platform metabolomics

Global metabolomes of oral swabs, stool and plasma were acquired by OE Biotech (project DZOE2024081068) on a dual platform comprising UPLC-MS/MS and GC-MS. A pooled quality-control sample was prepared from equal aliquots of all study samples. Raw data were processed with Progenesis QI v3.0 and MS-DIAL v4.24 [21], and metabolite annotations were matched against HMDB, LipidMaps, METLIN and vendor libraries [22–24]. Features were retained after relative-standard-deviation filtering, missing-value handling, log2 transformation and an identification-score threshold; annotation confidence was recorded using Metabolomics Standards Initiative levels [25]. Exogenous features were excluded from retained case-control analyses. Exogenous dexmedetomidine contamination precluded oral-metabolome case-control comparison, so the oral metabolome was excluded from the retained case-control and compartment-level analyses.

### Statistical analysis and multi-omics integration

All analyses were performed in R v4.3.2. Continuous variables were compared with the Wilcoxon rank-sum or Kruskal-Wallis test and categorical variables with Fisher's exact test; tests were two-sided. Unless otherwise stated, multiplicity was controlled with the Benjamini-Hochberg (BH) false-discovery rate [11], applied within pre-specified testing families as detailed below.

#### Microbiome diversity and differential abundance

Alpha diversity (Shannon, Chao1, Simpson) was computed with the vegan package [26]. Beta diversity used the Aitchison distance (Euclidean distance on centred-log-ratio [CLR]-transformed abundances) to respect the compositional nature of the data [10]; group differences were tested by PERMANOVA (adonis2, 999 permutations) with a companion multivariate dispersion test (betadisper) to distinguish location from dispersion effects. Differential abundance was assessed by Wilcoxon tests on CLR-transformed features with BH correction within each compartment, with effect sizes reported as the rank-biserial correlation. KEGG level-3 pathways were compared analogously (Wilcoxon, rank-biserial effect size, BH). Before differential testing, species were filtered to those present in at least 10% of samples within a compartment, retaining 1,324 of 3,013 oral and 1,079 of 3,463 faecal species; no abundance threshold was applied. Because 2bRAD-M yields marker-derived relative abundances rather than read counts, count-based differential-abundance frameworks such as ALDEx2 and ANCOM-BC were not applicable, and a compositional centred-log-ratio approach combined with rank-based testing was used instead. The 10% threshold was set a priori to limit the multiplicity burden imposed by ultra-rare taxa; sensitivity analyses applying no filter and thresholds of 5%, 20% and 30% are reported in Table S7. All species-level analyses used the 63 oral and 58 faecal 2bRAD-M samples. Before centred-log-ratio transformation, zeros were replaced by half the smallest non-zero relative abundance in the corresponding sample.

#### Metabolomics analysis

Group-level structure was visualised by OPLS-DA, whose robustness was assessed by response permutation testing (overfitting was indicated when permuted pR2Y/pQ2Y were non-significant). Per-feature differences used Wilcoxon tests with BH correction. Class-level metabolite-set enrichment analysis (MSEA) was performed over ClassyFire/chemical classes using MetaboAnalyst [27,28] to provide aggregated, class-level comparisons.

#### Clinical imaging analyses

The primary clinical analysis compared affected and contralateral alveolar bone density within each patient using two-sided paired Wilcoxon signed-rank tests. Associations of total alveolar bone loss with OMENS-Plus score, OMENS-M grade and Pruzansky-Kaban type were examined using Spearman correlation or Kruskal-Wallis testing, as appropriate. These imaging analyses were prespecified as descriptive clinical characterisation and are reported with their complete sample sizes and test statistics in Figure 1, Supplementary Figure S1 and Table S1.

#### Multiple-testing philosophy and reproducibility

Given the high dimensionality and collinearity of multi-omics data, nominal *P* values and Benjamini-Hochberg (BH) q values are supplied for every tested feature in the relevant supplementary tables. Main conclusions rely on FDR-controlled results or on the prespecified paired compartment test. The retained analyses specify the tested feature universe, inclusion criteria, transformation, model or test, multiplicity family and sample size in the Methods, figure legends and source tables. Analyses that did not meet these criteria were not retained as biological findings.

#### Formal testing of compartment specificity and statistical power

Differences in the number of FDR-significant features between compartments were not themselves treated as evidence of a compartment difference. Three complementary analyses were used. First, among the 58 participants with paired oral and gut 2bRAD-M profiles (41 CFM, 17 controls), globally z-scored Shannon diversity was modelled as value ~ group * compartment + (1 | participant); the group-by-compartment interaction was tested by likelihood-ratio comparison with the additive model. Second, the oral shotgun metagenome was repeatedly subsampled 1,000 times to the faecal shotgun sample size and case-control ratio (13 CFM, 9 controls), and the number of BH-significant pathways was compared with the observed faecal value. Third, we simulated location shifts to estimate the minimum absolute rank-biserial correlation detectable with 80% power at nominal alpha = 0.05 and at an FDR-equivalent threshold. We use ‘power-matched’ only for the oral and gut species-level analyses, which have the same minimum detectable effect; it describes statistical sensitivity and does not establish biological absence of a gut effect. The functional analyses are not power-matched and are interpreted as inconclusive for compartment specificity.

## Results

### Cohort characterisation and quantitative alveolar bone loss in craniofacial microsomia

#### A multi-omics CFM cohort with OMENS-Plus phenotyping

We assembled a multi-omics cohort of 45 patients with craniofacial microsomia (CFM) and 18 age- and sex-matched unaffected controls, recruited between October 2022 and April 2023 at the Plastic Surgery Hospital, Chinese Academy of Medical Sciences, Peking Union Medical College (approval no. 2021–199). Demographic and clinical characteristics are summarised in Table 1, with no significant between-group difference in age or sex. Available data layers per participant comprised oral and faecal microbiomes profiled by 2bRAD-M (oral *n* = 63; faecal *n* = 58), oral and faecal shotgun metagenomes (oral *n* = 48, of which 43 had matched clinical data; faecal *n* = 22), and blood and faecal metabolomes (blood *n* = 43; faecal *n* = 22); the complete availability matrix is shown in Figure 1A. Disease severity was graded with the OMENS-Plus system across the orbital (O), mandibular (M), ear (E), facial-nerve (N) and soft-tissue (S) domains (Figure 1E,F), and facial-nerve branch involvement was recorded in each patient (Figure 1G). Clinically, all patients had mandibular involvement (Pruzansky-Kaban IIa 48.9%, IIb 46.7%, III 4.4%), the median OMENS-Plus total score was 6.5 (mean 7.0 +/− 1.8; Figure 1F), and facial-nerve involvement was present in 20 of 45 patients (44.4%), with cranial-nerve extension to the hypoglossal (12/45, 26.7%) and trigeminal (2/45, 4.4%) nerves (Figure 1E,G; Table 1).

#### Alveolar bone loss is quantifiable and decoupled from composite severity

Because alveolar bone deficiency is a defining but under-quantified feature of CFM, we measured cortical bone density on 128-slice spiral computed tomography for the affected and contralateral sides in 35 patients. Density was lower on the affected side for the inner plate (paired Wilcoxon *P* = 3.8 × 10^−7), outer plate (*P* = 8.4 × 10^−4) and total alveolar bone (*P* = 9.7 × 10^−7; Figure 1B–D). Total bone loss was not associated with the OMENS-Plus total score (Spearman rho = 0.12, *P* = 0.493), OMENS-M grade (Kruskal-Wallis *P* = 0.64) or Pruzansky-Kaban type (*P* = 0.81; Supplementary Figure S1; Table S1). These results indicate that the paired imaging measure captures an aspect of alveolar involvement not represented by the categorical staging systems examined.

### Oral microbial and functional dysbiosis in CFM

#### Oral diversity and community composition differ between groups

We first examined the oral microbiome, the compartment most proximal to the craniofacial malformation. Oral Shannon diversity was higher in CFM than in controls (Wilcoxon *P* = 0.014, rank-biserial r = −0.40; Figure 2A), with concordant Chao1 and Simpson results (Supplementary Figure S2C and D; Table S2). Oral community composition also differed by group (Aitchison PERMANOVA *P* = 0.013; Figure 2B), without evidence of unequal multivariate dispersion (betadisper *P* = 0.231; Supplementary Figure S2A). At species level, four GTDB species clusters were lower in CFM after BH correction: Streptococcus mitis_I, Streptococcus mitis_AW, Granulicatella elegans and Granulicatella elegans_A (Figure 2D; Table S2). The present data establish differences in relative abundance, not that these taxa are universal markers of oral health or causal mediators of disease. The phylum-level composition is shown descriptively in Figure 2C. Analytical concordance of Streptococcus mitis and Granulicatella elegans across 2bRAD-M and shotgun metagenomic workflows is shown in Supplementary Figure S4.

#### Oral metagenomic pathway differences require non-disease-specific interpretation

We next analysed oral shotgun metagenomes at the KEGG pathway level. Of 429 annotated level-3 pathways, 209 met the prespecified eligibility criteria and 38 differed between groups after BH correction; 21 belonged to categories interpretable for an oral bacterial community and are displayed in Figure 2E, while the remaining 17 are reported without biological interpretation in Table S3. The displayed pathways include phosphotransferase-system and ABC-transport annotations, glycan and galactose metabolism, and cell-surface or biofilm-related processes. These functions describe substrate acquisition, carbohydrate use and cell-envelope biology; they can occur in health-associated as well as disease-associated oral organisms. Accordingly, the result is reported as a difference in inferred pathway abundance rather than evidence of a cariogenic, pathogenic or biofilm-dominant phenotype.

#### Patient-only post hoc hygiene, dietary and dental analyses

The recorded hygiene, dietary and dental items were available only in patients and were analysed post hoc. Within this limited subset, the retained oral pathway pattern was not associated with tooth-brushing frequency, sugary or carbonated drink intake, or caries count after the prespecified correction framework (Table S6). These analyses do not compare patients with controls, used a non-validated questionnaire and have limited power; they reduce neither the possibility of residual behavioural confounding nor the need for prospective measurement of diet, feeding and oral-health exposures.

### Evidence for an oral-predominant pattern

#### Gut microbiome and blood/faecal metabolomes show no FDR-robust case-control signature

We next examined the gut microbiome and the blood and faecal metabolomes. Gut Shannon diversity did not differ between groups (Wilcoxon *P* = 0.163, rank-biserial r = 0.24; Figure 3A), and the gut Aitchison PERMANOVA did not reach the conventional threshold (*P* = 0.072; betadisper *P* = 0.897; Figure 3B; Table S2). No blood or faecal chemical class survived BH correction in class-level metabolite-set enrichment analysis (minimum q = 0.05 and 0.11, respectively; Figure 3C; Table S5). These null findings are not interpreted as proof of biological absence, particularly for the smaller faecal shotgun subset. At the individual-feature level, no faecal metabolite survived BH correction, and OPLS-DA permutation testing did not support model validity (Supplementary Figure S3; Table S4).

**Figure 3.**
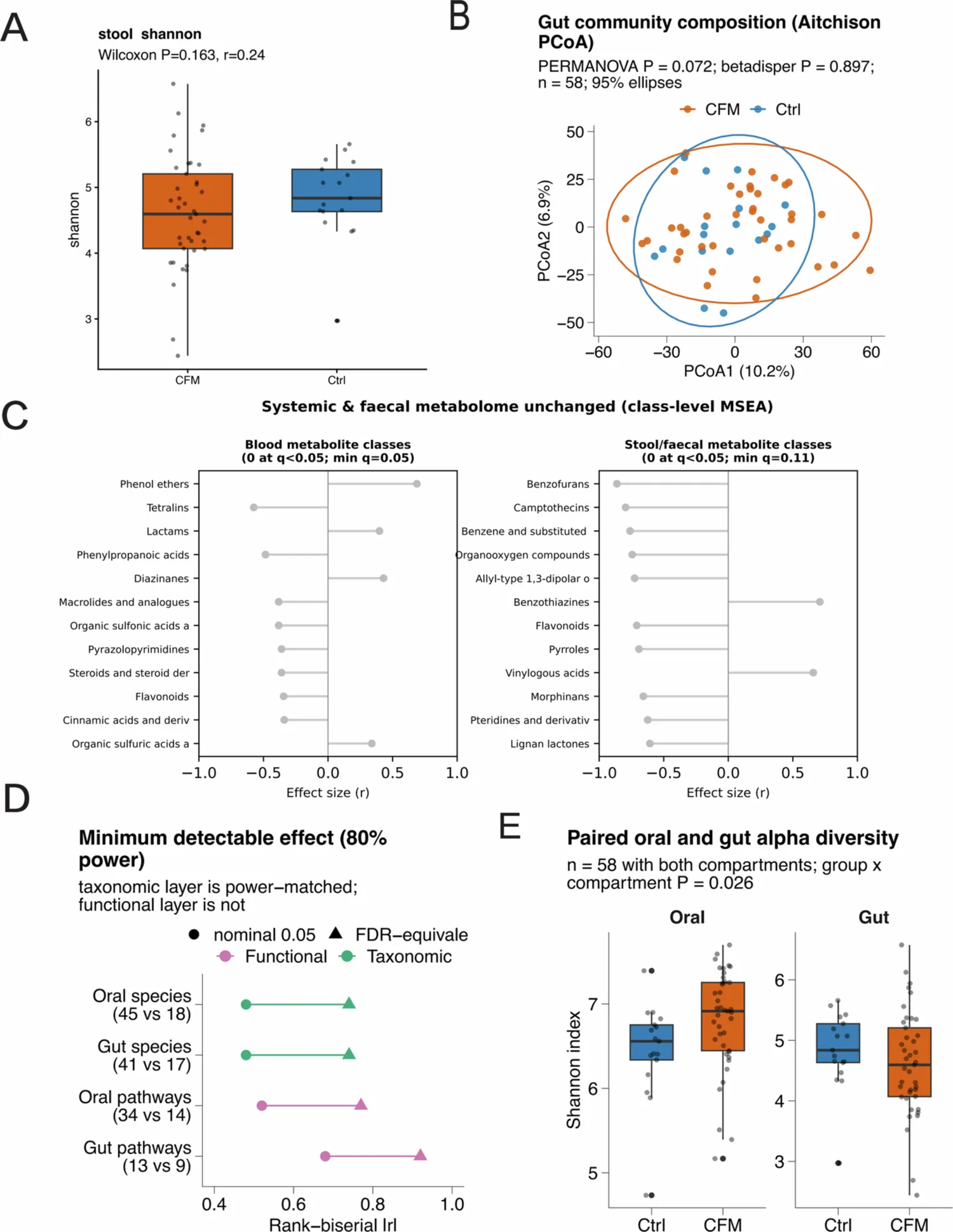
Compartment-resolved case-control analyses and statistical sensitivity. (A) Gut Shannon diversity in CFM versus controls (*n* = 58; 41 CFM, 17 controls); *P* is from a two-sided Wilcoxon rank-sum test and r is the rank-biserial correlation. (B) Principal coordinates analysis of gut Aitchison distance; ellipses are 95% confidence regions. *P* values are from PERMANOVA and betadisper. (C) Class-level metabolite-set enrichment analysis of blood (left) and faecal (right) metabolomes. No class survived BH-FDR in either compartment; the minimum q values are 0.05 and 0.11, respectively. (D) Minimum detectable absolute rank-biserial correlation at 80% power for oral species, gut species, oral pathways and gut pathways, at nominal alpha = 0.05 (circles) and the FDR-equivalent alpha for the relevant test family (triangles). The oral and gut species analyses are power-matched; the functional analyses are not. ‘Power-matched’ denotes comparable statistical sensitivity and does not establish biological absence of a gut effect. (E) Paired oral and gut Shannon diversity in the 58 participants with both profiles. Boxplots display the compartment-specific Shannon values; the group-by-compartment *P* value is from the prespecified likelihood-ratio test of globally z-scored Shannon diversity. Source statistics for panels A–E are in Tables S2, S5 and S8.

#### Species-level power matching supports an oral-predominant taxonomic pattern; functional compartment specificity remains inconclusive

At the species level, the oral (45 CFM, 18 controls) and gut (41 CFM, 17 controls) 2bRAD-M analyses had identical minimum detectable effects at 80% power (absolute rank-biserial r = 0.48 at nominal alpha and 0.74 at the FDR-equivalent threshold; Figure 3D; Table S8). In the 58 participants with paired profiles, the group-by-compartment interaction for globally z-scored Shannon diversity was significant (beta = 0.471; likelihood-ratio chi-square = 4.95, df = 1, *P* = 0.026; Figure 3E; Table S8). Together, these results support an oral-predominant taxonomic pattern under comparable sensitivity. They do not establish that a gut effect is biologically absent. Functional analyses were not power-matched: the FDR-equivalent minimum detectable effect was 0.77 for oral pathways and 0.92 for gut pathways. When oral shotgun data were subsampled to the faecal sample size and case-control ratio, the median number of significant pathways was 7, but 33.9% of 1,000 subsamples yielded none, matching the observed faecal value of 0 (empirical *P* = 0.34; Supplementary Figure S6; Table S8). The functional contrast is therefore inconclusive for compartment specificity.

## Discussion

### Overview of principal findings

In this multi-omics study of craniofacial microsomia (CFM), three observations are retained. First, oral diversity, community composition, four species-level taxa and a set of shotgun-derived pathway abundances differed between CFM and controls. Second, the species-level oral and gut comparisons were power-matched, and paired analysis supported an oral-predominant taxonomic pattern; neither this test nor the null gut comparisons prove biological absence of gut involvement. The functional comparison was not power-matched. Third, affected-side alveolar bone density was lower and the quantitative bone-loss measure was decoupled from the anatomical staging systems assessed. All findings arise from a cross-sectional observational design and describe associations rather than mechanisms or causal effects.

### Interpreting oral taxonomic and functional differences conservatively

Higher oral alpha diversity in CFM reproduces the direction reported in the earlier saliva-based CFM survey [8], but diversity is not itself a universal health metric. In contrast to the previous framing, our data do not show that higher richness represents opportunistic colonisation, nor do they establish that the four depleted GTDB clusters are health-associated commensals. Oral ecological diversity can vary with sampling site, host anatomy, maturation, caries activity, hygiene, diet and the taxa present; the current cross-sectional comparison cannot separate these contributors. We therefore report the direction of the diversity and abundance differences without assigning health, pathogenicity or causal status to them. Likewise, the observed KEGG differences include carbohydrate transport, glycan processing and cell-surface/biofilm-related annotations, but such functions are not specific to cariogenic communities and can support fitness of health-associated peroxigenic streptococci. The functional findings should be read as comparative metagenomic annotations, not as evidence of a pathogenic biofilm state.

### Interpreting oral predominance within the limits of the design

The compartment analysis is deliberately asymmetric in evidential strength. At species level, oral and gut analyses had the same minimum detectable effect and the paired group-by-compartment interaction was significant, supporting an oral-predominant taxonomic pattern. This use of ‘power-matched’ concerns statistical sensitivity only; it does not establish a biological absence of a gut effect. At the functional level, the faecal shotgun sample (13 patients, 9 controls) could detect only very large effects, and the downsampling analysis showed that a result of zero significant pathways is compatible with an oral effect of the observed magnitude. We therefore make no functional claim of compartment specificity. The lack of FDR-robust blood, faecal-metabolite and gut-taxonomic signals contrasts with the oral-gut-systemic spread described in periodontitis [9], but the cross-sectional single-centre design, unmeasured diet and limited distal-compartment samples preclude an inference that CFM is biologically compartment-restricted.

### Quantitative alveolar bone loss as a complementary clinical measure

The paired CT analysis provides a quantitative readout of alveolar involvement that was lower on the affected side and did not track the OMENS-Plus total score, OMENS-M grade or Pruzansky-Kaban type. This decoupling does not imply that imaging is a surrogate for clinical severity; rather, it suggests that the measure captures information not represented by the categorical anatomical schemes assessed here. Its clinical value and longitudinal responsiveness require prospective evaluation before it can be recommended as an endpoint.

### Diet, feeding and oral-health exposures remain important alternative explanations

Diet is a major determinant of faecal microbiome composition, and feeding or masticatory asymmetry may be particularly relevant in CFM. Apart from exclusion criteria and a patient-only sugary-drink item, these exposures were not measured. The post hoc patient-only analyses cannot determine whether dietary or oral-health variables explain the case-control oral findings, and they cannot rule out diet as a confounder or mediator of distal-compartment variation. Future studies should prospectively measure diet, feeding function, caries activity, periodontal status, oral-hygiene behaviour and sampling-site characteristics in both groups.

### Strengths and limitations

Strengths include species-resolved oral and gut profiling, the integration of blood and faecal metabolomics, paired CT-based assessment of alveolar bone density, and explicit control of compositionality and multiplicity. The study is nevertheless cross-sectional, single-centre and modest in size for distal-compartment and subgroup analyses. Controls were recruited from children undergoing minor elective scar-revision surgery rather than the community. Oral-hygiene, dietary and dental variables were patient-only and post hoc, and their sample sizes could not exclude modest confounding. Metabolite annotations span identification-confidence levels, and the functional analysis was underpowered in the faecal shotgun subset. The final interpretation therefore distinguishes FDR-controlled results, power-matched taxonomic comparisons and underpowered functional comparisons, and does not infer causality, pathogenicity or intervention efficacy.

## Conclusions

In summary, CFM was associated with oral taxonomic and functional microbiome differences, alongside reduced affected-side alveolar bone density that was not captured by the anatomical staging systems assessed. Species-level oral and gut analyses were power-matched and the paired diversity analysis supported an oral-predominant taxonomic pattern; this does not establish biological absence of a gut effect. The functional contrast remains underpowered and inconclusive for compartment specificity. Prospective, longitudinal work should measure diet, feeding, oral-health exposures and local anatomical variables, confirm candidate taxa and functions in independent cohorts, and test whether local, biofilm-directed interventions can improve clinically relevant outcomes.

## Supplementary Material

Supplementary figure legends Supplementary figure legends.docx

Table S6.xlsx Table S6.xlsx

Table S8.xlsx Table S8.xlsx

Table S1.xlsx Table S1.xlsx

Supplementary Figure S2F.tif Supplementary Figure S2F.tif

Supplementary Figure S1F.tif Supplementary Figure S1F.tif

Table S4.xlsx Table S4.xlsx

Table S7.xlsx Table S7.xlsx

Supplementary Figure S3F.tif Supplementary Figure S3F.tif

Table S3.xlsx Table S3.xlsx

Supplementary Figure S6F.tif Supplementary Figure S6F.tif

Supplementary Figure S4F.tif Supplementary Figure S4F.tif

Supplementary Figure S5F.tif Supplementary Figure S5F.tif

Table S5.xlsx Table S5.xlsx

Table S2.xlsx Table S2.xlsx

## Data Availability

The data that support the findings of this study are openly available. The 2bRAD-M and shotgun metagenomic sequencing data are deposited in the NCBI BioProject repository under accession PRJNA1118189 (https://www.ncbi.nlm.nih.gov/bioproject/PRJNA1118189). The untargeted metabolomics data (blood, faecal and oral) are deposited in the OMIX database of the National Genomics Data Center, China National Center for Bioinformation, under accession OMIX017122 (https://ngdc.cncb.ac.cn/omix/release/OMIX017122). For the retained analyses, the supplementary materials provide the tested feature universe, sample inclusion, transformation, statistical test, multiplicity framework and machine-readable result tables. De-identified clinical and imaging-derived variables, R analysis scripts, package versions and figure-to-output mapping information are available from the corresponding author (X.T.; Frog30@126.com) on reasonable request, subject to institutional policies governing paediatric patient data. A list of abbreviations used in this manuscript is provided in Appendix 1.

## Appendix 1. List of abbreviations

**Table:**

ABC	ATP-binding cassette
ACME	average causal mediation effect
BH	Benjamini-Hochberg
BH-FDR	Benjamini-Hochberg false-discovery rate
CFM	craniofacial microsomia
CLR	centred log-ratio
CT	computed tomography
FDR	false-discovery rate
GABA	gamma-aminobutyric acid
GC-MS	gas chromatography-mass spectrometry
HU	Hounsfield units
KEGG	Kyoto Encyclopedia of Genes and Genomes
MSEA	metabolite-set enrichment analysis
MSI	Metabolomics Standards Initiative
OMENS	orbit, mandible, ear, nerve, soft tissue (classification)
OMENS-Plus	OMENS classification with extracranial anomalies
OPLS-DA	orthogonal partial least-squares discriminant analysis
PERMANOVA	permutational multivariate analysis of variance
RDA	redundancy analysis
RSD	relative standard deviation
UPLC-MS/MS	ultra-performance liquid chromatography-tandem mass spectrometry
X-Index	amino-acid biosynthetic-catabolic gene-content balance index
2bRAD-M	type IIB restriction-site associated DNA sequencing for microbiome
