# Supplementary material for: Oral-predominant microbial and functional dysbiosis in craniofacial microsomia: species-level profiling with compartment-matched statistical testing
Source: J Oral Microbiol. 2026 Nov 3;18(1):2754580. doi: 10.1080/20002297.2026.2754580 (PMC13757820; doi:10.1080/20002297.2026.2754580)
Supplement: Supplementary file 1 [file ZJOM_A_2754580_SM0814.docx]

**Supplementary figure legends**

Supplementary Figure S1. Alveolar bone loss and anatomical severity. (A) Correlation between OMENS-Plus total score and total alveolar bone loss in CFM patients (n = 35). (B) Inner, outer and total bone loss stratified by OMENS-M grade. (C) Bone loss stratified by Pruzansky-Kaban type. (D) OMENS-Plus total score excluding its facial-nerve component, stratified by neural-involvement category (n = 45). (E) Distribution of total alveolar bone loss. P values are from Spearman correlation or Kruskal-Wallis testing as appropriate; full statistics are in Table S1.

Supplementary Figure S2. Robustness of diversity estimates and bone-loss distributions. (A-B) Multivariate dispersion for oral and gut communities; P values are permutation-test (betadisper) results. (C-D) Chao1 and Simpson diversity in the oral compartment. (E-F) Chao1 and Simpson diversity in the gut compartment. (G-H) Inner- and outer-plate bone-loss distributions. Diversity comparisons use two-sided Wilcoxon rank-sum tests. This figure supports the main diversity analyses and does not establish a health or pathogenicity interpretation for diversity.

Supplementary Figure S3. Faecal metabolome quality and feature-level case-control analysis. (A) OPLS-DA scores for the faecal metabolome (n = 22; 8,089 features). (B) Response permutation testing of the OPLS-DA model. (C) Feature-level volcano plot; no feature survived BH-FDR q < 0.05, and highlighted points are nominal P < 0.05 only. (D) Distribution of Metabolomics Standards Initiative annotation-confidence levels. Source statistics are in Table S4.

Supplementary Figure S4. Analytical concordance of two taxonomic-assignment workflows for retained oral taxa. Relative abundance of (A) Streptococcus mitis and (B) Granulicatella elegans measured by 2bRAD-M and shotgun metagenomics in the same 48 oral DNA extracts (34 CFM, 14 controls). Both axes are shown on a log(1+x) relative-abundance scale; P values are two-sided Spearman tests. The platforms use distinct sequencing and taxonomic-assignment workflows but share the same DNA extract. The comparison therefore demonstrates analytical concordance, not independent biological replication.

Supplementary Figure S5. Per-participant data availability. Availability of each data type for 63 participants (45 CFM, 18 controls), ordered by completeness within group. Blue indicates available and grey indicates not collected. Columns represent oral and faecal 2bRAD-M, oral and faecal shotgun metagenomes, oral, faecal and blood metabolomes, CT-derived alveolar bone loss, inflammatory markers, and oral-hygiene/dental variables. Bone loss, inflammatory markers and hygiene/dental variables were patient-only measures. Aggregate counts are shown in Figure 1A and Table S1.

Supplementary Figure S6. Oral shotgun-metagenome subsampling to the faecal sample size and case-control ratio. In 1,000 random oral subsamples of 13 CFM and 9 controls, the median number of BH-FDR-significant KEGG level-3 pathways was 7. The observed faecal value was 0; 339 of 1,000 oral subsamples (33.9%) also yielded 0 significant pathways, giving an empirical P value of 0.34. This sensitivity analysis shows that the functional comparison was not power-matched and is inconclusive for compartment specificity. Source statistics are in Table S8.
